# Seroprevalence of CMV IgG and IgM in Korean women of childbearing age

**DOI:** 10.1002/jcla.23716

**Published:** 2021-03-30

**Authors:** Rihwa Choi, Sukjung Lee, Sang Gon Lee, Eun Hee Lee

**Affiliations:** ^1^ Department of Laboratory Medicine Green Cross Laboratories Yongin Korea; ^2^ Department of Laboratory Medicine and Genetics Samsung Medical Center Sungkyunkwan University School of Medicine Seoul Korea; ^3^ Green Cross Laboratories Yongin Korea

**Keywords:** congenital infection, cytomegalovirus, maternal screening, seroprevalence

## Abstract

**Background:**

The aim of this study was to investigate the seroprevalence of cytomegalovirus (CMV) infection using the serologic status of CMV IgG and IgM antibodies in Korean women of childbearing age.

**Methods:**

We retrospectively reviewed CMV IgG and IgM test results from Korean women aged 15–49 years who underwent testing between January 2009 and December 2019. Seroprevalence of CMV IgG and IgM by year and age was investigated.

**Results:**

The study period was 11 years, and among 6837 samples tested, 95.8% were CMV IgG–positive. The seropositivity in women aged 15‐ <20 years was significantly lower (77.5%) than older age groups. Among 6837 total CMV IgG tests, 75.9% (5186) had concurrently measured CMV IgM results among which 2.4% were IgM‐positive.

**Conclusion:**

Considering the low CMV seropositivity of women younger than 20 years, they may need prenatal education for CMV infection.

## 1 INTRODUCTION

Cytomegalovirus (CMV) is a human herpes virus affecting 66%–90% of adults worldwide.^1^ While the primary infection is often asymptomatic, CMV establishes a lifelong latent infection that can become active in both immunocompetent and immunosuppressed carriers.^2^ CMV infection during pregnancy can result in congenital infection of the foetus.^2^ The range of disease severity of congenital CMV infection is very wide, from normal development to sensorineural hearing loss, chorioretinitis, and cognitive or neurologic deficits that may be mild or severe.^2^, ^3^

For diagnosis of CMV infection, serology tests, such as CMV IgG and IgM, can be used.^2^, ^4^ Seroepidemiologic studies (seropositivity) using these tests can provide information on disease control because individuals with no history of exposure to CMV infection may be at high risk of primary infection, and seropositive individuals can experience reactivation of latent CMV.^1^ Recent studies have reported changes in global seroepidemiology of CMV based on geographic, ethnic, cultural and socioeconomic factors.^1^, ^5^, ^6^ Previous studies' different ethnic populations used different analytical platforms and different seroprevalence of CMV over different study periods.^1^, ^5^ However, limited data are available for CMV seroprevalence in the Korean population.^7^, ^8^

Previous studies performed in Korean women include only CMV IgG seroprevalence without CMV IgM seroprevalence or information on age of subjects, which is important in CMV seroprevalence; data analysed were from earlier than 2015; or there were a limited number of study subjects.^1^, ^6^, ^8^ Therefore, we aimed to investigate recent seroprevalence of CMV IgG and IgM test results in Korean women overall and by year and age in this study.

## 2 MATERIALS AND METHODS

We retrospectively reviewed the laboratory information system data from Green Cross Laboratories between 1 January 2009 and 31 December 2019 to investigate seroprevalence of CMV IgG. Green Cross Laboratories, one of the largest referral clinical laboratories in South Korea, provides clinical specimen analysis services including CMV IgG and CMV IgM chemiluminescence immunoassays (Architect i2000SR; Abbott Laboratories). A ‘reactive (+)'result interpretation for the CMV IgG assay was any with ≥6.0 AU/ml. For qualitative interpretation of the CMV IgM assay, a ‘reactive (+)’ result was defined as that with ≥1.00 index, a ‘non‐reactive (−)’ result was that with <0.85 index, and ‘grey zone’ results were for 0.85–0.99 index.

Patients with missing data for age or sex were excluded. Because the aim of this study was to investigate seroprevalence in Korean women of childbearing age, repetitive test results were excluded. All data were anonymized prior to statistical analysis. A public database for annual numbers of patients with congenital CMV infection in Korea was reviewed through Healthcare Bigdata Hub by Health Insurance Review & Assessment Service (HIRA) using the 10th revision, Clinical Modification of the International Statistical Classification of Diseases and Related Health Problems (ICD‐10‐CM) Code P35.1 for congenital CMV infection (available at: http://opendata.hira.or.kr/op/opc/olap4thDsInfo.do). The study protocol was approved by the Institutional Review Board (IRB) of Green Cross Laboratories (GCL‐2020–1046–01). Because the study was retrospective and involved no more than minimal risk to the subjects, a waiver of informed consent was approved by the IRB. The study was conducted in accordance with the Declaration of Helsinki.

Numbers and percentages of each utilized test are presented as seropositivity and seronegativity of CMV IgG and IgM. Non‐parametric analyses were used when the data were not normally distributed. The chi‐square and Mann‐Whitney tests were used when appropriate to compare results in year and age groups. The Spearman correlation analysis was performed to investigate the association between the annual rate of ‘non‐reactive’ results of CMV IgG and that of ‘reactive’ or ‘grey zone’ results of CMV IgM. *P*‐values less than 0.05 were considered significant. Statistical analyses were executed using MedCalc Statistical Software version 19.1.5 (MedCalc Software bv, Ostend, Belgium; https://www.medcalc.org; 2020).

## 3 RESULTS

**FIGURE 1:**
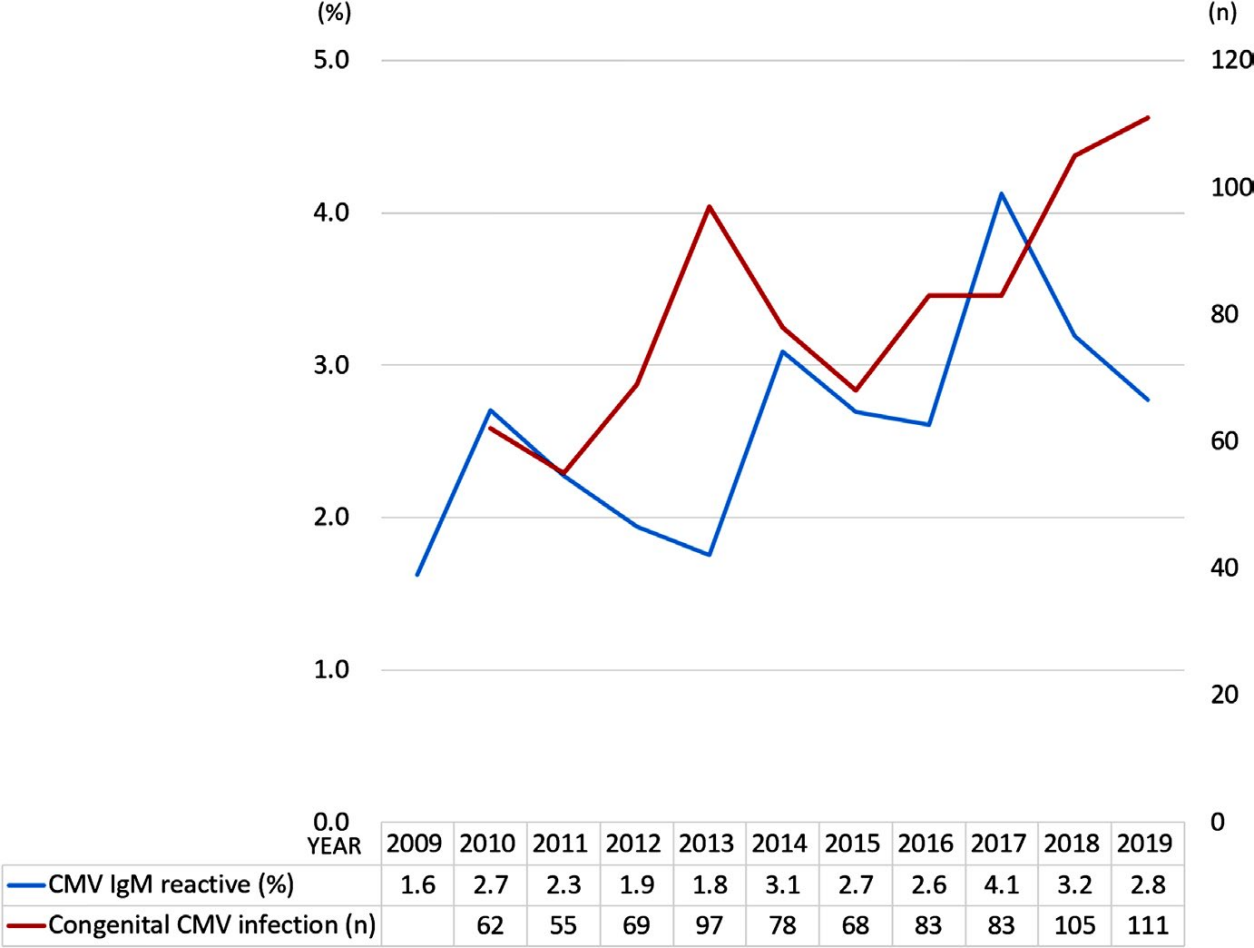
CMV IgM seroprevalence among Korean women of childbearing age and number of congenital CMV infections (ICD‐10‐CM P35.1) by year. Left vertical axis is CMV IgM seroprevalence, and right vertical axis is number of congenital CMV infections. Number of congenital CMV infections in 2009 was not available in the database

During the 11‐year study period, 7436 CMV IgG tests from 6837 Korean women aged 15–49 years were obtained from the laboratory information system of Green Cross Laboratories. After exclusion of repetitive measurements, 6837 CMV IgG tests from Korean women with a median age of 32.3 years (interquartile range 28.4–37.5 years) were included. The overall rate of ‘reactive’ results for CMV IgG (seropositivity) among 6837 CMV IgG test results was 95.8%. Annual numbers of requested CMV IgG test were significantly different (*p* < 0.05). Number of CMV IgG test requests was highest in 2011 (1082 tests) and lowest in 2017 (357 tests). The rate of ‘non‐reactive’ results for CMV IgG (seronegativity) by year was significantly different (*p* < 0.01) and ranged from 1.8% in 2011 to 7.7% in 2014 (Figure 1). There was negative association between total number of requested CMV IgG and rate of ‘non‐reactive’ CMV IgG (*r* = −0.74, *p* < 0.01). The rate of ‘non‐reactive’ results for CMV IgG by age group was significantly different, and women aged 15‐ <20 years showed the largest proportion of ‘non‐reactive’ results (22.5%, *p* < 0.05).

Among 6837 CMV IgG test results, 75.9% (5186) had concurrently measured CMV IgM results. The CMV IgG and IgM test results of 5186 subjects are summarized in Table 1. Seroprevalence of CMV IgG and IgM in Korean women by age and year is summarized in Table 2. Annual changes in the rate of ‘non‐reactive’ CMV IgG and ‘reactive’ or ‘grey zone’ CMV IgM are shown in Table 2. Annual rate of ‘non‐reactive’ CMV IgG and ‘reactive’ or ‘grey zone’ CMV IgM was significantly associated (*r* = 0.77, *p* < 0.01). Annual number of patients managed for congenital CMV infection (ICD‐10‐CM P35.1) between 2010 and 2019 as reported on Healthcare Bigdata Hub by HIRA is presented in Figure 1. There was no association between number of congenital CMV infection cases and the rate of ‘non‐reactive’ CMV IgG or CMV IgM results such as the rate of ‘reactive’ or ‘reactive or grey zone’ (*p* ≥ 0.05).

**TABLE 1:** Qualitative results of CMV IgG and IgM tests

		CMV IgG		
		Non‐reactive	Reactive	Total
CMV IgM	Non‐reactive	196^a^	4833^b^	5029
	Grey zone	0	30^c^	30
	Reactive	6^d^	121^e^	127
	Total	202	4984	5186

^a^ IgG‐/IgM‐ (4.8%) is suggestive of no CMV infection, patient who might need prenatal education for CMV infection, and/or repeat test or further action needed if there is clinical suspicion of recent infection.

^b^ IgG+/IgM‐ is suggestive of past infection, or need for repeat test or further tests to differentiate recent primary infection/reactivation/past infection based on clinical findings.

^c^ IgG+/IgM grey zone (0.6%) results that might require further tests to differentiate between recent primary infection/reactivation/past infection.

^d^ IgG‐/IgM+ (0.1%) may suggest recent primary infection (early state before IgG increase after infection)

^e^ IgG+/IgM+ (2.3%) indicates need for further tests to differentiate between recent primary infection/reactivation/past infection.

**TABLE 2:** Seroprevalence of CMV IgG and IgM in Korean women by age and year

Year	2009	2010	2011	2012	2013	2014	2015	2016	2017	2018	2019	Total
CMV IgG												
15‐<20 years, total (n)	19	25	14	20	25	26	18	17	10	6	1	181
Non‐reactive (n)	4	7	3	3	3	10	7	6	3	1	0	47
Reactive (n)	15	18	11	17	22	16	11	11	7	5	1	134
CMV IgG reactive (%)	78.9	72.0	78.6	85.0	88.0	61.5	61.1	64.7	70.0	83.3	100.0	74.0
20‐<30 years, total (n)	185	185	309	194	190	146	94	53	77	106	120	1659
Non‐reactive (n)	5	2	8	4	12	19	9	3	16	17	6	101
Reactive (n)	180	183	301	190	178	127	85	50	61	89	114	1558
CMV IgG reactive (%)	97.3	98.9	97.4	97.9	93.7	87.0	90.4	94.3	79.2	84.0	95.0	93.9
30‐<40 years, total (n)	223	246	513	359	291	194	136	97	73	184	252	2568
Non‐reactive (n)	5	2	2	3	8	6	1	2	0	3	13	45
Reactive (n)	218	244	511	356	283	188	135	95	73	181	239	2523
CMV IgG reactive (%)	97.8	99.2	99.6	99.2	97.3	96.9	99.3	97.9	100.0	98.4	94.8	98.2
40‐49 years, total (n)	66	62	87	97	65	55	49	63	58	80	96	778
Non‐reactive (n)	0	1	0	1	0	3	1	0	0	2	1	9
Reactive (n)	66	61	87	96	65	52	48	63	58	78	95	769
CMV IgG reactive (%)	100.0	98.4	100.0	99.0	100.0	94.5	98.0	100.0	100.0	97.5	99.0	98.8
Overall, total (n)	493	518	923	670	571	421	297	230	218	376	469	5186
Non‐reactive (n)	14	12	13	11	23	38	18	11	19	23	20	202
Reactive (n)	479	506	910	659	548	383	279	219	199	353	449	4984
CMV IgG reactive (%)	97.2	97.7	98.6	98.4	96.0	91.0	93.9	95.2	91.3	93.9	95.7	96.1
CMV IgM												
15‐<20 years, total (n)	19	25	14	20	25	26	18	17	10	6	1	181
Non‐reactive (n)	18	22	13	17	19	22	17	15	9	4	1	157
Grey zone (n)	0	0	0	1	0	2	0	0	0	0	0	3
Reactive (n)	1	3	1	2	6	2	1	2	1	2	0	21
CMV IgM reactive (%)	5.3	12.0	7.1	10.0	24.0	7.7	5.6	11.8	10.0	33.3	0.0	11.6
20‐<30 years, total (n)	185	185	309	194	190	146	94	53	77	106	120	1659
Non‐reactive (n)	184	179	299	189	186	139	91	50	73	102	115	1607
Grey zone (n)	0	2	1	0	2	0	0	1	1	2	0	9
Reactive (n)	1	4	9	5	2	7	3	2	3	2	5	43
CMV IgM reactive (%)	0.5	2.2	2.9	2.6	1.1	4.8	3.2	3.8	3.9	1.9	4.2	2.6
30‐<40 years, total (n)	223	246	513	359	291	194	136	97	73	184	252	2568
Non‐reactive (n)	218	239	503	354	289	190	131	96	71	179	248	2518
Grey zone (n)	2	1	2	0	0	1	2	0	0	2	1	11
Reactive (n)	3	6	8	5	2	3	3	1	2	3	3	39
CMV IgM reactive (%)	1.3	2.4	1.6	1.4	0.7	1.5	2.2	1.0	2.7	1.6	1.2	1.5
40‐49 years, total (n)	66	62	87	97	65	55	49	63	58	80	96	778
Non‐reactive (n)	62	61	83	95	64	53	48	62	55	74	90	747
Grey zone (n)	1	0	1	1	1	1	0	0	0	1	1	7
Reactive (n)	3	1	3	1	0	1	1	1	3	5	5	24
CMV IgM reactive (%)	4.5	1.6	3.4	1.0	0.0	1.8	2.0	1.6	5.2	6.3	5.2	3.1
Overall, total (n)	493	518	923	670	571	421	297	230	218	376	469	5186
Non‐reactive (n)	482	501	898	655	558	404	287	223	208	359	454	5029
Grey zone (n)	3	3	4	2	3	4	2	1	1	5	2	30
Reactive (n)	8	14	21	13	10	13	8	6	9	12	13	127
CMV IgM reactive (%)	1.6	2.7	2.3	1.9	1.8	3.1	2.7	2.6	4.1	3.2	2.8	2.4

## 4 DISCUSSION

We investigated seroprevalence of CMV IgG and IgM in Korean women of childbearing age (15–49 years) over 11 years. The overall seroprevalence in Korean women was 95.8%, higher than the global CMV seroprevalence of 86.0%.^1^ The rate of positive CMV IgG was lowest (less than 80%) in women younger than 20 years. Previous studies performed in Korean women reported that overall CMV IgG seroprevalence was 98.1% in pregnant women investigated in 2008^6^ and 97.0% in women of childbearing age (15–49 years) investigated during 1995–2015.^8^ In a later study, the seroprevalence of CMV IgG in women aged 15–19 years was 83.8%, comparable to that in the present study.^8^ In the present study, CMV IgG seronegativity by year ranged from 1.8% to 7.7% (Figure 1). This is a large difference that is most likely due to the age make up of women tested in a given year (Table 2). Meanwhile, for CMV IgM, there are few studies regarding seroprevalence in Korean women.^7^, ^8^ Previous studies regarding CMV IgM seroprevalence were performed in different ethnic populations using different analytical platforms, such as enzyme‐linked immunosorbent assay (ELISA), chemiluminescence immunoassay (CLIA) and chemiluminescent microparticle immunoassay (CMIA) test kits to test reported various ranges of seroprevalence.^1^ In the present study, overall CMV IgM seropositivity was 2.4%, with the highest seroprevalence of 11.6% in women aged 15‐ <20 years. This high seropositivity rate was comparable to those in previous studies including those of young pregnant women in India (overall 13.63% and 16.4% in the 15‐ to 25‐year age group)^9^ and Africa (ranged from 0% to 15.5% through systematic review of the literature).^10^ A previous study performed in the United States using NHANES III 1998–1994 reported overall 5.3% CMV IgM prevalence in 12‐ to 19‐year‐old women.^11^ Overall CMV IgM seroprevalence in the present study (2.4%) was comparable to that of other studies performed in Korea, Norway and the United States.^5^, ^7^, ^11^ Difference in seroprevalence might be due to differences in pre‐analytical (ethnic and cultural differences) and analytical factors (analytical platform).^1^, ^5^

The incidence of congenital CMV infection among previously seronegative pregnant women in the United States ranges from 0.7% to 4.0%.^12^ A previous study performed in the United States described that the majority of pregnant women in their study did not have knowledge of CMV.^3^ Meanwhile, studies regarding the seropositivity of CMV and the risk of having a child with congenital CMV infection or patient perception of CMV infection among women of childbearing age or pregnant in Korea are limited. In South Korea, routine CMV screening using CMV IgG or IgM in women of childbearing age and/or pregnant is not recommended by national public health bodies. Considering that women who are seronegative in their first pregnancy have higher risk of CMV infection and risk of giving birth to a child with congenital CMV infection in their subsequent pregnancies than women in the general population, education and prevention strategies for mothers in this age group may be needed.^2^, ^13^

In this study, about 3.0% of test results were IgG+/IgM+ (2.3%), IgG‐/IgM+ (0.1%), or IgG+/IgM grey zone (0.6%), indicating subjects requiring further laboratory tests to distinguish primary CMV infection from past CMV infection/reactivation.^4^ A previous study performed in pregnant Korean women reported rates of CMV IgG+/IgM+1.3% and CMV IgG+/IgM grey zone 0.4% comparable to the present study.^7^ A previous study performed in pregnant Japanese women reported IgG+/IgM+ (4.3%), IgG‐/IgM+ (0.2%) and IgG+/IgM grey zone (1.2%), rates about two times higher than those of the present study.^14^

Although there was no significant difference between annual number of congenital CMV infection and CMV IgG or IgM results in this study, the number of congenital CMV infections was more than five to 10 times higher than that of congenital rubella infection, with fewer than 10 annual cases under nationwide disease control with an immunization programme.^6^ However, the efficacy of a national health screening programme for CMV infection still needs to be investigated.^4^, ^14^, ^15^ Future studies regarding the efficacy of CMV screening in large populations are needed.

One limitation of this study was the lack of clinical information, such as symptoms of infection. However, because 25%–50% of pregnant women with primary CMV infection are asymptomatic, serologic tests can provide important information.^2^, ^4^ Seroprevalence studies may help to estimate the burden of disease attributable to congenital CMV infection.^1^, ^4^ The strength of this study was the large study population analysed over a long study period. The results of this study could be used as basic knowledge to support disease control and prevention of congenital CMV infection. Gynaecologists and general practitioners should propose CMV serology tests in pregnant women and/or young women of childbearing age (especially <20 years) who develop influenza‐like symptoms (fever, fatigue and headache) not attributable to another specific infection.^2^ Furthermore, this study may help to identify populations at higher risk of having a child with congenital CMV infection that require further education.^3^

In conclusion, this study investigated seroprevalence of CMV IgG and IgM in Korean women of childbearing age. Considering the rate of seronegative CMV IgG in women younger than 20 years, education and prevention for young mothers may be needed. The results of this study could be used as foundational knowledge for strengthening disease control and prevention of congenital CMV infection.

## CONFLICT OF INTEREST

None declared.

## AUTHOR CONTRIBUTIONS

All authors contributed to manuscript preparation; R. Choi, S. Lee, S.G. Lee and E. H. Lee collected the data or contributed to data analysis; R. Choi and S.G. Lee designed the study; R. Choi, S.G. Lee and E. H. Lee had full access to all the data in the study and takes responsibility for the integrity of the data and the accuracy of the data analysis. All authors read and approved the final manuscript.

## Data Availability

The datasets generated and analysed during the current study are available from the corresponding authors on reasonable request.
